# Carotid Intima–Media Thickness and Narrowing in Rheumatoid Arthritis: Impact of Age, Diabetes, and Conventional Risk Factors

**DOI:** 10.3390/biomedicines14040817

**Published:** 2026-04-03

**Authors:** Fahad F. Almutairi, Jaber H. Alsalah, Husam K. Alzubaidi, Mohammad Mustafa, Yasser Bawazir, Khalid Bahamdein, Hassan Balubaid, Haifa Alnahdi, Khalid Khashoggi, Bander Almutairi, Shoaa Shetewi, Fahad Alrwaithi, Shadi Abushaheen, Dana Attraji, Sara Alsaylani, Huda Alamri, Rawan Abdeen, Hamzah H. Ahmed, Mohammad Khalil

**Affiliations:** 1 Department of Radiologic Sciences, Faculty of Applied Medical Sciences, King Abdulaziz University, Jeddah 21589, Saudi Arabia; jhalyami@kau.edu.sa (J.H.A.); falrowaithi@stu.kau.sa (F.A.); sabushaheen0001@stu.edu.sa (S.A.); daatarji@stu.edu.sa (D.A.); salsylani@stu.kau.edu.sa (S.A.); habdulrahmanalamri@stu.edu.sa (H.A.); aeabdeen@kau.edu.sa (R.A.); hhahmed@kau.edu.sa (H.H.A.); 2 Radiology Unit, King Fahd Medical Center, King Abdulaziz University, Jeddah 21589, Saudi Arabia; 3 Department of Medicine, University of Jeddah, Jeddah 21589, Saudi Arabia; mamustafa@uj.edu.sa; 4 Department of Medicine, King Abdulaziz University, Jeddah 21589, Saudi Arabia; ymbawazir@kau.edu.sa; 5 Department of Internal Medicine, King Saud University, Riyadh 11421, Saudi Arabia; 6 Department of Internal Medicine and Rheumatology, East Jeddah Hospital, Ministry of Health, Jeddah 11176, Saudi Arabia; hkbalubaid@moh.gov.sa; 7 Department of Radiology, Faculty of Medicine, King Abdulaziz University, Jeddah 21589, Saudi Arabia; hmalnahdi@kau.edu.sa (H.A.); kkhashoggi@kau.edu.sa (K.K.); balmutairi@kau.edu.sa (B.A.); sshetewi@kau.edu.sa (S.S.); mrkhalil@kau.edu.sa (M.K.)

**Keywords:** rheumatoid arthritis, subclinical atherosclerosis, carotid intima–media thickness, cardiovascular risk, diabetes mellitus

## Abstract

**Background:** Rheumatoid arthritis (RA) has been linked to increased cardiovascular risk; however, whether RA independently contributes to subclinical atherosclerosis remains unclear. This study aimed to evaluate carotid intima–media thickness (IMT) and carotid narrowing in RA patients compared with controls and to examine their associations with conventional cardiovascular risk factors. **Methods:** A total of 73 RA patients and 78 healthy controls underwent carotid Doppler ultrasonography to assess IMT and carotid narrowing. Non-parametric analyses were used for between-group comparisons, and associations with clinical variables were evaluated within the RA cohort. **Results:** The median age was 55 years (IQR: 43–63) in the RA group and 61 years (IQR: 51–68) in controls (*p* = 0.012). No significant differences were observed in median right CCA IMT (RA: 0.070 mm [IQR: 0.060–0.081] vs. controls: 0.068 mm [IQR: 0.050–0.076]; *p* = 0.619) or left CCA IMT (RA: 0.065 mm [IQR: 0.051–0.079] vs. controls: 0.065 mm [IQR: 0.050–0.074]; *p* = 0.701). The prevalence of carotid narrowing was also comparable between groups (right CCA: 15.1% vs. 11.5%, *p* = 0.633). Within the RA cohort, age was positively correlated with right CCA IMT (Spearman’s rho = 0.375, *p* = 0.001), and diabetes mellitus was associated with a higher prevalence of right CCA narrowing (34.8% vs. 6.0%, *p* = 0.003). **Conclusions:** Carotid IMT and narrowing were not significantly different between RA patients and controls. In this cohort, age and diabetes mellitus were more strongly associated with subclinical carotid atherosclerosis than RA status itself. These findings emphasize the importance of comprehensive cardiovascular risk assessment in RA patients, particularly focusing on traditional risk factors.

## 1. Introduction

Rheumatoid arthritis (RA) is a long-standing autoimmune condition characterized by persistent synovial inflammation, chronic joint deterioration, and lasting disability [1,2]. In recent years, there has been increasing recognition that RA is not confined to the joints but also confers a markedly elevated risk of cardiovascular disease (CVD), which now represents a major cause of morbidity and mortality in affected individuals [3,4,5]. Epidemiological studies indicate that patients with RA have up to a 50% higher risk of developing CVD events compared to the general population, even after adjustment for traditional cardiovascular risk factors such as hypertension, diabetes mellitus, and smoking [6,7,8].

The mechanisms underlying the increased cardiovascular risk in RA are multifactorial, involving traditional risk factors, persistent systemic inflammation, disease activity, and the impact of disease-modifying antirheumatic drugs (DMARDs) [8,9,10]. Chronic inflammation in RA has been linked to atherosclerosis changes through endothelial dysfunction, oxidative stress, and alterations in lipid metabolism, which together may contribute to subclinical vascular changes [4,11,12].

Intima–media thickness (IMT) and plaque detection via carotid Doppler ultrasonography are validated, non-invasive markers of subclinical atherosclerosis, indicating the presence of vascular involvement and linked to increased cardiovascular risk [5,10,13]. Several studies have shown increased IMT and a higher number of plaques in RA populations compared to controls, although there remains debate over the extent to which these changes are attributable to RA itself versus conventional cardiovascular risk factors [7,14,15,16].

Given the increased cardiovascular risk and potential for early atherosclerotic changes in RA, there is a clear need for early identification and intervention to mitigate long-term vascular complications [2,17,18]. However, there is a lack of local data regarding subclinical atherosclerosis in RA populations.

This study aims to explore the prevalence of subclinical carotid atherosclerosis, as assessed by Doppler ultrasonography, in patients with RA compared to healthy controls. Furthermore, the study seeks to identify the associations between traditional cardiovascular risk factors, RA-related variables, and ultrasound-based markers of atherosclerosis.

## 2. Methods

### 2.1. Design and Setting

This cross-sectional study was performed at the Radiology Department, King Abdulaziz University Hospital, between March 2023 and August 2023. The study aimed to evaluate subclinical atherosclerosis in RA patients compared to healthy controls, using carotid Doppler ultrasonography.

### 2.2. Ethical Considerations

The study was approved by the Ethics Committee of the Faculty of Medicine, King Abdulaziz University Hospital (Approval No. 501-22). Informed consent was obtained from all participants prior to enrolment.

### 2.3. Participants

A number of 151 participants were enrolled in the study, comprising 73 patients diagnosed with RA and 78 healthy individuals as controls. Controls were recruited during the same study period from hospital staff and community volunteers. Formal matching for age, sex, or smoking status was not performed. Consequently, differences in baseline characteristics were addressed analytically using multivariable regression models adjusting for age, sex, and smoking status. No propensity score matching was performed. Inclusion criteria for the RA group were a confirmed diagnosis of RA according to the ACR (American College of Rheumatology) criteria and age ≥ 18 years [19]. The control group consisted of healthy volunteers with no history of autoimmune disease. Exclusion criteria included a history of other autoimmune or chronic inflammatory diseases, previous cardiovascular disease or significant carotid artery pathology, pregnancy, and incomplete clinical or ultrasound data.

### 2.4. Data Collection

Demographic and clinical data were obtained from all participants, including age, gender, height, weight, body mass index (BMI), smoking status, and histories of diabetes mellitus, hypertension, and stroke. Laboratory parameters such as complete blood count, urea, creatinine, C-reactive protein (CRP), erythrocyte sedimentation rate (ESR), hemoglobin, rheumatoid factor (RF), and anti-cyclic citrullinated peptide (anti-CCP) antibodies were recorded for the RA group. Inflammatory status within the RA cohort was assessed using routinely available laboratory markers, including C-reactive protein (CRP) and erythrocyte sedimentation rate (ESR). Standardized disease activity indices such as the Disease Activity Score in 28 joints (DAS28), cumulative corticosteroid exposure, and specific pro-inflammatory cytokine measurements (e.g., TNF-α, IL-6, IL-1β) were not incorporated into the study protocol.

### 2.5. Carotid Ultrasonography

All participants underwent bilateral carotid Doppler ultrasonography using a high-resolution ultrasound system equipped with a linear probe. Participants were scanned in supine position with the head slightly turned to the contralateral side to optimize visualization. Both longitudinal and transverse planes were scanned to assess the common carotid arteries (CCA) wall. IMT measurements were obtained from the far wall of the common carotid artery approximately 1 cm proximal to the carotid bifurcation during longitudinal scanning. To reduce measurement variability, five IMT measurements were obtained for each segment, and the average value was used for analysis. All examinations were performed by experienced sonographers following a standardized scanning protocol to ensure measurement consistency.

### 2.6. Statistical Analysis

Statistical analyses were performed using IBM SPSS Statistics (version 26) and R software (version 4.4.1). Continuous variables were summarized as medians and interquartile ranges (IQR), and categorical variables as counts and percentages. Normality of continuous variables was assessed using the Shapiro–Wilk test (Supplementary Materials Table S1). As several variables deviated from normality, non-parametric tests were used for between-group comparisons. Continuous variables were compared using the median test, while categorical variables were analyzed using the chi-square test or Fisher’s exact test, as appropriate. Correlations were assessed using Spearman’s rho.

## 3. Multivariable Modeling

To examine the independent association between rheumatoid arthritis (RA) status and carotid IMT, multivariable linear regression models were constructed, adjusting for age, sex, and smoking status. Regression diagnostics (Supplementary Materials Table S2) indicated heteroskedasticity and non-normal residuals; therefore, robust regression using MM-estimation was applied. For carotid narrowing outcomes, logistic regression models were constructed, adjusting for age, sex, and smoking status. Given the limited number of narrowing events (20 right-sided, 5 left-sided, and 22 combined events), events-per-variable (EPV) ratios were below the recommended threshold of 10 in several models (Supplementary Materials Table S3). To address potential small sample bias and separation, Firth’s penalized logistic regression was used. Given the number of subgroups and regression analyses performed, these analyses were considered exploratory. No formal correction for multiple comparisons was applied. A two-tailed *p*-value < 0.05 was considered statistically significant.

## 4. Results

A total of 73 patients with rheumatoid arthritis (RA) and 78 healthy controls were included in this study. The demographic and clinical characteristics of the participants are summarized in Table 1. The RA group comprised 58 females (79.45%) and 15 males (20.55%), whereas the control group included 35 females (44.87%) and 43 males (55.13%). The median age was significantly lower in the RA group (55 years, IQR: 43–63) compared with controls (61 years, IQR: 51–68; *p* = 0.012). Median height was also significantly lower among RA patients (157 cm, IQR: 153–161.25) compared with controls (164.5 cm, IQR: 157–170.25; *p* < 0.001). In addition, smoking prevalence was significantly lower in the RA group (2.74%) than in controls (12.82%; *p* = 0.032). No significant differences were observed between groups in weight (*p* = 0.192) or BMI (*p* = 0.862). Within the RA cohort, the median disease duration was 72 months (IQR: 24.5–168). The prevalence of diabetes mellitus (DM) was 31.51%, hypertension (HTN) 28.77%, stroke 4.17%, and ischemic heart disease (IHD) 6.94% (Table 1). Laboratory parameters are detailed in Table 1, including a median CRP of 4.85 mg/L and ESR of 22 mm/h. Rheumatoid factor (RF) positivity was observed in 53.85% of patients, and anti-CCP antibodies in 39.29%.

Analysis of carotid intima–media thickness (IMT) and the presence of carotid intima–media thickness (IMT) measurements are presented in Table 2. No statistically significant differences were observed in median right CCA IMT between RA patients (0.07 mm, IQR: 0.06–0.0805) and controls (0.068 mm, IQR: 0.05–0.07625; *p* = 0.619). Similarly, left CCA IMT did not differ significantly between groups (0.065 mm in both groups; *p* = 0.701).

Multivariable linear regression models adjusting for age, sex, and smoking did not initially demonstrate a significant independent association between RA status and carotid IMT. However, regression diagnostics indicated heteroskedasticity and non-normal residuals, which may violate assumptions of standard regression models. Therefore, robust regression using MM-estimation was applied to provide more reliable estimates under these conditions. In these models, RA status showed a modest association with right CCA IMT and mean IMT. This discrepancy between standard and robust models suggests that the observed association is sensitive to model assumptions and should be interpreted cautiously. To avoid selective emphasis, both conventional and robust regression results are presented, with primary emphasis placed on the overall consistency of findings and the between-group comparisons, which did not demonstrate significant differences in IMT. Accordingly, these findings are considered exploratory rather than confirmatory (Table 3).

### 4.1. IMT and Regression Diagnostics

Assessment of normality demonstrated significant deviation for left CCA IMT (Supplementary Materials Table S1). Regression diagnostics confirmed heteroskedasticity and non-normal residuals, justifying the use of robust MM-estimation (Supplementary Materials Table S2). In robust multivariable regression models (Table 3), RA status was independently associated with right CCA IMT and mean IMT after adjustment for age, sex, and smoking. Age remained significantly associated with right CCA IMT.

The prevalence of carotid narrowing is summarized in Table 4. Right CCA narrowing was present in 15.07% of RA patients and 11.54% of controls (*p* = 0.633). Left CCA narrowing occurred in 4.11% of RA patients and 2.56% of controls (*p* = 0.673). When considering either side, narrowing was observed in 16.44% of RA patients and 12.82% of controls (*p* = 0.646). These differences were not statistically significant.

Multivariable logistic regression adjusting for age, gender, and smoking (Table 5) confirmed that RA status was not independently associated with carotid narrowing (right side OR = 1.689, *p* = 0.335; left side OR = 1.505, *p* = 0.684; combined OR = 0.631, *p* = 0.374). Age was significantly associated with right-sided narrowing (OR = 1.049, *p* = 0.018).

### 4.2. Carotid Narrowing and Model Stability

The prevalence of carotid narrowing did not differ significantly between RA patients and controls (Table 4). Due to limited narrowing events and low EPV ratios (Supplementary Materials Table S3), Firth’s penalized logistic regression was applied. In adjusted models (Table 5), RA status was not independently associated with carotid narrowing. Age demonstrated a significant association with right-sided narrowing. Given the sparse event distribution, these findings should be interpreted cautiously.

Correlations between continuous variables and IMT within the RA group are presented in Table 6. Age showed a significant positive correlation with right CCA IMT (Spearman’s rho = 0.375, *p* = 0.001). No other continuous variables, including weight, BMI, laboratory markers, or RA duration, were significantly correlated with IMT.

Associations between categorical variables and IMT are shown in Table 7. No significant associations were found for gender, smoking, DM, HTN, stroke, IHD, RF, or anti-CCP status. However, the association between anti-CCP status and left CCA IMT approached statistical significance (*p* = 0.054).

Associations between categorical variables and narrowing are presented in Table 8. DM was significantly associated with right CCA narrowing (34.78% in diabetics vs. 6% in non-diabetics; *p* = 0.003) and combined narrowing (*p* = 0.007). No other categorical variables showed significant associations.

Comparisons of continuous variables between patients with and without narrowing are summarized in Table 9, Table 10 and Table 11. For right CCA narrowing (Table 9), patients with narrowing were significantly older (median 63 years vs. 55 years; *p* = 0.038). No significant associations were observed with BMI, RA duration, or laboratory markers. For left CCA narrowing (Table 10), no significant associations were identified. When considering narrowing on either side (Table 11), age showed a borderline association (*p* = 0.054).

## 5. Discussion

The aim of this cross-sectional study was to assess subclinical carotid atherosclerosis in patients with rheumatoid arthritis compared to healthy controls. To ensure balanced interpretation, the findings from both conventional and robust regression models are considered together, and greater emphasis is placed on results that are consistent across analytical approaches. The results did not demonstrate a statistically significant difference in carotid IMT or in the prevalence of carotid narrowing between RA patients and controls (Table 2 and Table 3). These results suggest that the presence of RA status was not independently associated with a greater burden of subclinical carotid atherosclerosis when controlling for conventional cardiovascular risk factors, which is in line with recent studies highlighting the importance of traditional risk factors such as age and diabetes mellitus. Conventional regression models did not demonstrate a significant association between RA status and carotid IMT. However, robust regression analysis identified a modest association with right CCA IMT and mean IMT after adjustment for confounders. These findings should be interpreted cautiously, given the exploratory nature of the analysis as well as the lack of independent association with carotid narrowing; further support for this observation can be found in Table 3 and Table 5.

The discrepancy between standard and robust regression findings highlights the importance of model selection and underlying assumptions in observational analyses. Robust regression was applied to address violations of normality and heteroskedasticity; however, such methods may yield different effect estimates compared to conventional approaches. Therefore, these results should not be interpreted as definitive evidence of an independent effect of RA, but rather as hypothesis-generating findings that warrant confirmation in larger and more rigorously controlled studies.

**Figure 1:**
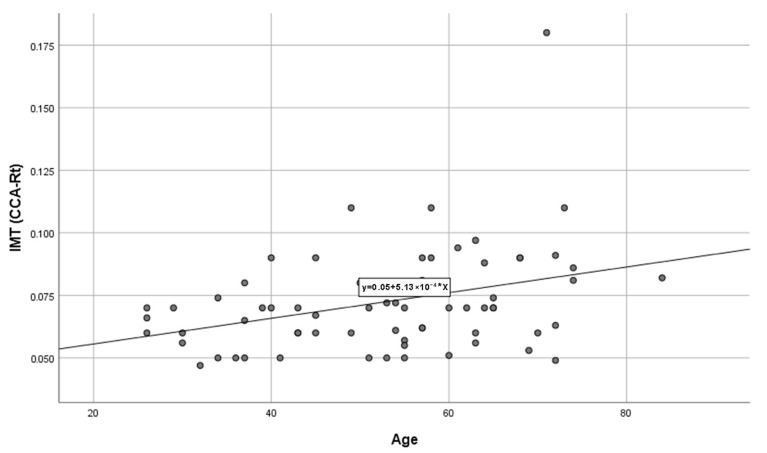
Shows significant positive correlation between age and right CCA IMT.

Age emerged as the only continuous variable independently associated with higher right CCA IMT in RA patients, a finding consistent with previous research demonstrating the importance of age as a determinant of early atherosclerotic changes both in the general population and among individuals with RA [4,5]. The observed positive correlation between age and IMT (Table 6, Figure 1) reinforces the need to account for age-related vascular changes when evaluating cardiovascular risk in this population.

Diabetes mellitus was found to be a significant determinant of carotid narrowing in RA patients, with diabetic individuals showing a markedly higher prevalence of right CCA narrowing (Table 8). Additional subgroup analysis demonstrated that patients with right CCA narrowing were significantly older than those without narrowing (Table 9), and age showed a borderline association when narrowing on either side was considered (Table 11). This association is consistent with previous studies identifying diabetes as a critical risk factor for the development of atherosclerotic vascular changes in both RA and non-RA cohorts [7,8]. The current findings further emphasize that, while systemic inflammation and disease activity in RA may contribute to cardiovascular risk, the impact of metabolic comorbidities such as diabetes is substantial and appears to have a greater association than RA characteristics.

Although earlier reports have suggested an increased prevalence of subclinical atherosclerosis in RA patients attributed to chronic systemic inflammation and immune dysregulation [2,12] the lack of a statistically significant difference in IMT and carotid narrowing between groups in this study (Table 2 and Table 4) supports recent evidence that traditional cardiovascular risk factors remain the most reliable predictors of subclinical atherosclerosis in RA. The association between anti-CCP antibody status and left CCA IMT approached statistical significance (Table 7), which is consistent with the hypothesis that RA seropositivity may be linked to vascular pathology, though this requires confirmation in studies with larger sample sizes.

Several limitations should be acknowledged. First, the cross-sectional design of the study precludes causal inferences regarding the relationships between rheumatoid arthritis (RA), cardiovascular risk factors, and subclinical atherosclerosis. In addition, the single-center nature of the study and the demographic characteristics of the sample may limit the generalizability of the findings.

Another limitation relates to the incomplete assessment of certain cardiovascular risk factors. Although several conventional risk factors, such as diabetes mellitus, hypertension, smoking status, age, and sex, were included in the analysis, additional determinants, including lipid profile, physical activity, family history of cardiovascular disease, and the use of medications such as statins or antihypertensive agents, were not systematically evaluated. Furthermore, information regarding the duration and level of control of diabetes or hypertension was not available. The absence of these variables may introduce residual confounding and limit the ability to fully distinguish the relative contributions of traditional cardiovascular risk factors versus RA-related mechanisms in the development of subclinical atherosclerosis.

Inflammatory burden was evaluated using routine laboratory markers (CRP and ESR); however, standardized RA disease activity indices (e.g., DAS28), cumulative corticosteroid exposure, and cytokine profiling (e.g., TNF-α, IL-6, IL-1β) were not measured. Therefore, the mechanistic evaluation of inflammation-driven vascular changes was limited. Although disease duration and selected laboratory markers were analyzed within the RA cohort, comprehensive inflammatory characterization was not available, and RA was primarily evaluated as a binary exposure in between-group comparisons rather than being stratified by disease activity. Given the heterogeneity of RA and its potential influence on cardiovascular risk, this may limit the depth of RA-specific characterization.

The primary vascular outcomes assessed in this study were carotid intima–media thickness (IMT) and the presence of carotid narrowing. Resistance index (RI) measurements and ankle-brachial index (ABI) assessments were not included in the ultrasound protocol. Although carotid IMT and narrowing are validated markers of subclinical atherosclerosis, the inclusion of additional vascular indices could provide complementary information regarding systemic vascular involvement.

RA patients were managed according to routine clinical practice rather than a standardized research protocol, resulting in variability in treatment regimens depending on disease activity and physician discretion. Treatment-related variables, including biologic therapy exposure, were not systematically incorporated into adjusted models and may therefore represent a source of residual confounding.

The relatively small number of carotid narrowing events, particularly for left-sided narrowing, may have limited statistical power and affected model stability despite the use of Firth’s penalized logistic regression. Although events-per-variable (EPV) ratios were calculated and penalized methods were applied to mitigate small-sample bias, some models may still have been underpowered and prone to both type I and type II errors. Accordingly, effect estimates—particularly those with wide confidence intervals—should be interpreted with caution.

In addition, the RA and control groups were not formally matched for age and sex, resulting in baseline imbalance. Although multivariable adjustment was performed, analytical correction does not fully substitute for matched design or propensity score adjustment. Therefore, residual confounding cannot be excluded, particularly given the recruitment of controls from hospital staff and community volunteers, the absence of detailed cardiovascular variables (e.g., lipid profile, medication use, physical activity), and the lack of comprehensive RA-specific measures such as disease activity indices and treatment exposure. These factors may influence both vascular outcomes and group comparisons, and therefore, the findings should be interpreted within the context of these limitations.

## 6. Conclusions

In this cross-sectional study, carotid IMT and carotid narrowing were not significantly different between patients with rheumatoid arthritis and healthy controls. Within the RA cohort, age and diabetes mellitus were more consistently associated with markers of subclinical atherosclerosis. Although exploratory analyses suggested a potential association between RA status and IMT in robust models, this finding was not consistent across analytical approaches and should be interpreted cautiously. Given the observational design, baseline imbalance between groups, and limited number of outcome events, analytical adjustment may not fully account for these differences; therefore, these findings should be interpreted as hypothesis-generating rather than definitive evidence. Larger, longitudinal studies with comprehensive cardiovascular and disease-specific characterization are needed to further clarify these relationships.

## Figures and Tables

**Table 1:** Sample Characteristics and Demographics.

Variables/Type of Statistic Used	Estimated Statistic Within the RA-Sample	Estimated Statistic Within the Healthy-Sample	*p*-Value *
Median (IQR) for age	55 (43–63)	61 (51–68)	0.012
N (%) for males	15 (20.55%)	43 (55.13%)	0.000
N (%) for females	58 (79.45%)	35 (44.87%)	
N (%) for smokers	2 (2.74%)	10 (12.12.82%)	0.032
Median (IQR) for weight (kg)	76 (63–85)	78.5 (69.75–86)	0.192
Median (IQR) for height (cm)	157 (153–161.25)	164.5 (157–170.25)	0.000
Median (IQR) for BMI	28.71 (25.27–35.1)	28.53 (24.99–32.91)	0.862
Median (IQR) for months of RA	72 (24.5–168)		
N (%) of DM	23 (31.51%)		
N (%) of HTN	21 (28.77%)		
N (%) of Stroke	3 (4.17%)		
N (%) of IHD	5 (6.94%)		
Median (IQR) for CBC (million/m^3^)	10.8 (6.87–12.3)		
Median (IQR) for urea (mmol/L)	3.9 (3–4.6)		
Median (IQR) for creatine (µmol/L)	57 (50–69.75)		
Median (IQR) for CRP (mg/L)	4.85 (3.19–13.58)		
Median (IQR) for ESR (mm/h)	22 (12.75–36)		
N (%) of positive RF	21 (53.85%)		
N (%) of positive anti-CCP	11 (39.29%)		
Median (IQR) for Hb (g/dL)	10.8 (6.87–12.3)		

Note. Whenever there were missing cases, the percentages were calculated based on the adjusted total sample size (after exclusion of the missing cases). * Result of chi-square test (for the categorical variables, i.e., gender and smoking) and median test (for the continuous variables, i.e., age, weight, height and BMI).

**Table 2:** Median (and IQR) of IMT in the RA and Healthy Samples.

Side (of CCA)	Sample		*p*-Value
	RA	Healthy	
Right	0.07 (0.06–0.0805)	0.068 (0.05–0.07625)	0.619
Left	0.065 (0.0505–0.079)	0.065 (0.05–0.07425)	0.701

**Table 3:** The Multivariate Association between RA and IMT, with Confounders (Age, Gender, and Smoking) being Controlled.

DV	IV	B	SE	*p*-Value	95%—CI	R^2^
Rt. CCA-IMT	(Constant)	0.03887	0.00576	0.001	0.02729–0.0493	0.166
	RA	0.00799	0.00280	0.005	0.00277–0.01357	
	Age	0.00040	0.00010	0.001	0.00022–0.0006	
	Gender	0.00341	0.00298	0.135	−0.00223–0.00947	
	Smoking	−0.00224	0.00424	0.310	−0.01117–0.00545	
Lt. CCA-IMT	(Constant)	0.05367	0.00688	0.001	0.03991–0.06637	0.051
	RA	0.00343	0.00329	0.135	−0.00309–0.00983	
	Age	0.00012	0.00012	0.159	−0.0001–0.00035	
	Gender	0.00061	0.00314	0.399	−0.00553–0.00683	
	Smoking	0.01529	0.00585	0.012	0.00308–0.02619	
CCA-IMT (avg. of rt & lt)	(Constant)	0.04793	0.00545	0.001	0.0368–0.05822	0.078
	RA	0.00514	0.00252	0.021	0.00026–0.0101	
	Age	0.00026	0.00008	0.003	0.0001–0.00042	
	Gender	0.00042	0.00239	0.458	−0.00409–0.00537	
	Smoking	0.00343	0.00566	0.257	−0.00998–0.01253	

**Table 4:** Presence of Narrowing(s) in the RA and Healthy Samples.

Sample	Total N	Presence of Narrowing *n* (%)	*p*-Value
CCA-Rt			
Patients (RA)	73	11 (15.07%)	0.633
Healthy	78	9 (11.54%)	
CCA-Lt			
Patients (RA)	73	3 (4.11%)	0.673
Healthy	78	2 (2.56%)	
CCA—Any Side (Combined)			
Patients (RA)	73	12 (16.44%)	0.646
Healthy	78	10 (12.82%)	

**Table 5:** The Multivariate Association between RA and Presence of Narrowing, with Confounders (Age, Gender, and Smoking) being Controlled.

DV	IV	B	SE	OR	95%—CI of OR	*p*-Value	Overall *p*-Value
Presence of narrowing (CCA-Rt)	(Constant)	−5.019	1.335	0.007	0–0.082	0.000	0.123
	RA	0.493	0.516	1.637	0.586–4.714	0.347	
	Age	0.045	0.019	1.046	1.008–1.09	0.016	
	Gender	0.423	0.563	1.527	0.522–5.154	0.451	
	Smoking	0.319	0.972	1.376	0.129–8.095	0.753	
Presence of narrowing (CCA-Lt)	(Constant)	−4.540	1.965	0.011	0–0.533	0.020	0.957
	RA	0.341	0.834	1.406	0.228–9.577	0.709	
	Age	0.019	0.029	1.020	0.957–1.093	0.554	
	Gender	0.343	0.913	1.409	0.235–15.785	0.726	
	Smoking	0.270	1.518	1.310	0.009–20.082	0.872	
Presence of narrowing (any side)	(Constant)	−4.364	1.235	0.013	0.001–0.131	0.000	0.216
	RA	0.435	0.494	1.545	0.578–4.222	0.385	
	Age	0.038	0.018	1.039	1.003–1.079	0.031	
	Gender	0.303	0.530	1.354	0.489–4.166	0.569	
	Smoking	0.090	0.955	1.095	0.105–6.091	0.926	

**Table 6:** The Correlation between Continuous Characteristic Variables and IMT within the Sample of Patients.

Variable	Its Correlation with:			
	IMT (CCA-Rt)		IMT (CCA-Lt)	
	Spearman’s rho	*p*-Value	Spearman’s rho	*p*-Value
Age	0.375	0.001	0.209	0.076
Weight	−0.184	0.163	−0.030	0.821
Height	−0.181	0.175	−0.059	0.662
BMI	−0.095	0.477	0.003	0.981
CBC	−0.091	0.441	−0.103	0.385
Urea	0.077	0.522	−0.051	0.671
Creatinine	−0.118	0.323	0.041	0.731
CRP	0.054	0.655	0.080	0.502
ESR	0.086	0.481	0.063	0.607
Hb	−0.091	0.441	−0.103	0.385
Months of RA	0.124	0.330	0.055	0.663

**Table 7:** The Association between Categorical Characteristic Variables and IMT within the Sample of Patients.

Variable	Category	N	Mdn (IQR) for IMT (CCA-Rt)	*p*-Value	Mdn (IQR) for IMT (CCA-Lt)	*p*-Value
Gender	Male	15	0.07 (0.06–0.08)	0.610	0.067 (0.051–0.076)	0.953
	Female	58	0.07 (0.06–0.083)		0.065 (0.05–0.081)	
Smoker	Yes	2	0.06 (0.05–0)	0.612	0.066 (0.045–0)	0.486
	No	71	0.07 (0.06–0.081)		0.065 (0.051–0.078)	
DM	Yes	23	0.07 (0.057–0.09)	0.709	0.067 (0.055–0.081)	0.560
	No	50	0.07 (0.06–0.08)		0.063 (0.0495–0.077)	
HTN	Yes	21	0.08 (0.0585–0.084)	0.061	0.067 (0.0535–0.0795)	0.554
	No	52	0.07 (0.06–0.08)		0.063 (0.05–0.0793)	
Stroke	Yes	3	0.06 (0.05–0)	0.670 *	0.045 (0.045–0)	0.286 *
	No	69	0.07 (0.06–0.0805)		0.066 (0.053–0.0805)	
IHD	Yes	5	0.07 (0.0665–0.1305)	0.329 *	0.09 (0.061–0.0935)	0.072 *
	No	67	0.07 (0.06–0.08)		0.065 (0.051–0.077)	
RF	Negative	18	0.065 (0.06–0.08)	0.563	0.065 (0.0488–0.07)	0.415
	Positive	21	0.07 (0.068–0.089)		0.067 (0.05–0.0875)	
Anti-CCP	Negative	17	0.08 (0.06–0.085)	0.054	0.06 (0.045–0.07)	0.440
	Positive	11	0.07 (0.067–0.07)		0.087 (0.059–0.09)	

* Median test was replaced with Mann–Whitney test here; refer to the Section 2.

**Table 8:** The Association between Categorical Characteristic Variables and Narrowing within the Sample of Patients.

Variable	Category	N	Cases with Narrowing(s) by CCA Side:					
			Rt.	*p*-Value	Lt.	*p*-Value	Both Sides Combined	*p*-Value
Gender	Male	15	2 (13.33%)	1.000	0 (0%)	1.000	2 (13.33%)	1.000
	Female	58	9 (15.52%)		3 (5.17%)		10 (17.24%)	
Smoker	Yes	2	0 (0%)	1.000	0 (0%)	1.000	0 (0%)	1.000
	No	71	11 (15.49%)		3 (4.23%)		12 (16.9%)	
DM	Yes	23	8 (34.78%)	0.003	2 (8.7%)	0.232	8 (34.78%)	0.007
	No	50	3 (6%)		1 (2%)		4 (8%)	
HTN	Yes	21	4 (19.05%)	0.719	2 (9.52%)	0.197	5 (23.81%)	0.308
	No	52	7 (13.46%)		1 (1.92%)		7 (13.46%)	
Stroke	Yes	3	1 (33.33%)	0.397	0 (0%)	1.000	1 (33.33%)	0.426
	No	69	10 (14.49%)		3 (4.35%)		11 (15.94%)	
IHD	Yes	5	1 (20%)	0.575	1 (20%)	0.197	1 (20%)	1.000
	No	67	10 (14.93%)		2 (2.99%)		11 (16.42%)	
RF	Negative	18	2 (11.11%)	0.667	0 (0%)	N/A	2 (11.11%)	0.667
	Positive	21	4 (19.05%)		0 (0%)		4 (19.05%)	
Anti-CCP	Negative	17	4 (23.53%)	0.132	1 (5.88%)	1.000	4 (23.53%)	0.132
	Positive	11	0 (0%)		0 (0%)		0 (0%)	

**Table 9:** The Association between Continuous Characteristic Variables and Narrowing (in CCA-Rt) within the Sample of Patients.

Characteristic Variable	Median (IQR) for:		*p*-Value *
	Cases with Narrowing(s)	Cases Without Narrowing(s)	
Age	63 (50–71)	55 (40–61.25)	0.038
Weight (kg)	64 (62–75.8)	76 (65–85)	0.126
Height (cm)	154 (149–158)	157 (153–162)	0.108
BMI	27.89 (24.77–29.15)	29.48 (25.28–35.84)	0.445
Months of RA	84 (36–183)	72 (24–162)	0.433
CBC (million/m^3^)	10.9 (6.8–12.2)	10.75 (6.85–12.45)	0.799
Urea (mmol/L)	3.3 (3–4.5)	4 (2.95–4.6)	0.667
Creatinine (µmol/L)	53 (48–84)	57 (50–68.5)	0.845
CRP (mg/L)	3.9 (3.2–16.8)	5 (3.19–13.45)	0.962
ESR (mm/h)	22 (19–34)	22 (11–40)	0.366
Hb (g/dL)	10.9 (6.8–12.2)	10.75 (6.85–12.45)	0.799

* Mann–Whitney test was used.

**Table 10:** The Association between Continuous Characteristic Variables and Narrowing (in CCA-Lt) within the Sample of Patients.

Characteristic Variable	Median (IQR) for:		*p*-Value *
	Cases with Narrowing(s)	Cases Without Narrowing(s)	
Age	54 (49–54)	55 (42.5–63)	0.763
Weight (kg)	85 (62–85)	75.9 (63.25–82.75)	0.397
Height (cm)	154 (148–154)	157 (153–162)	0.342
BMI	35.84 (28.31–35.84)	28.53 (25.24–34.66)	0.218
Months of RA	84 (60–84)	72 (24–168)	0.587
CBC (million/m^3^)	6.8 (6.27–6.8)	10.85 (7.41–12.45)	0.239
Urea (mmol/L)	5.6 (2.6–5.6)	3.9 (3–4.55)	0.299
Creatinine (µmol/L)	80 (47–80)	57 (50–68.5)	0.326
CRP (mg/L)	16.8 (8.27–16.8)	4.4 (3.19–13.1)	0.093
ESR (mm/h)	35 (19–35)	22 (12–34)	0.355
Hb (g/dL)	6.8 (6.27–6.8)	10.85 (7.41–12.45)	0.239

* Mann–Whitney test was used.

**Table 11:** The Association between Continuous Characteristic Variables and Narrowing (in CCA, Lt and Rt combined) within the Sample of Patients.

Characteristic Variable	Median (IQR) for:		*p*-Value *
	Cases with Narrowing(s)	Cases Without Narrowing(s)	
Age	60.5 (51–70.25)	55 (40–61.5)	0.054
Weight (kg)	65.5 (62.25–82.7)	76 (65–85)	0.239
Height (cm)	154 (149.25–157.5)	157.5 (153–162)	0.082
BMI	28.1 (25.33–34.17)	29.39 (25.27–35.25)	0.659
Months of RA	83.5 (42–182.25)	72 (24–165)	0.529
CBC (million/m^3^)	9.85 (6.4–12.08)	10.8 (7.25–12.5)	0.527
Urea (mmol/L)	3.55 (3.03–5.33)	3.95 (2.93–4.6)	0.970
Creatinine (µmol/L)	53 (49–83)	57 (50–68)	0.844
CRP (mg/L)	6.09 (3.21–16.8)	4.85 (3.19–13.15)	0.705
ESR (mm/h)	25.5 (19.75–34.75)	20.5 (10.75–39.25)	0.236
Hb (g/dL)	9.85 (6.4–12.08)	10.8 (7.25–12.5)	0.527

* Mann–Whitney test was used.

## Data Availability

The data supporting the findings of this study are available from the corresponding author upon reasonable request.

## Supplementary Materials

The following supporting information can be downloaded at: https://www.mdpi.com/article/10.3390/biomedicines14040817/s1, Table S1: Normality of the Continuous Variables; Table S2: Normality of the Continuous Variables; Table S3: Number of Cases in All Possible Subgroups.
