# Supplementary material for: Carotid Intima–Media Thickness and Narrowing in Rheumatoid Arthritis: Impact of Age, Diabetes, and Conventional Risk Factors
Source: Biomedicines. 2026 Apr 3;14(4):817. doi: 10.3390/biomedicines14040817 (PMC13113294; doi:10.3390/biomedicines14040817)
Supplement: Supplementary file 1 [file biomedicines-14-00817-s001.zip › biomedicines-4146998-supplementary.pdf]

Appendix Table S1: Normality of the Continuous Variables

Variable	P-value of Shapiro-Wilk Test
Age	0.957
Weight	0.385
Height	0.000
BMI	0.000
Months of RA	0.024
CBC	0.041
Urea	0.020
Creatinine	0.238
CRP	0.000
ESR mm/hr	0.001
Anti-CCP	0.000
Hb g/dl	0.041
Rt CCA_IMT	0.162
Lt CCA_IMT	0.000

Note. P-value > .05 indicates non-significant deviation from normality; usually interpreted as an indication of normality. P-value < .05 indicates a significant deviation from normality; usually interpreted as an indication of non-normality.

Appendix Table S2: Normality of the Continuous Variables

Indicator	Linear Regression Model		
	Outcome = IMT (R)	Outcome = IMT (L)	Outcome = IMT (Avg.)
Max Cook's Distance (Outlier Indicator)	0.38	0.994	0.794
P-value of Breusch Pagan's Test (Heteroskedasticity Indicator)	0.004	< .001	< .001
P-value of Shapiro-Wilk's Test (Nonnormality Indicator)	< .001	< .001	< .001
VIF (Multicollinearity Indicator)	1.24 for RA; 1.09 for age; 1.26 for gender; 1.13 smoking		

Appendix Table S3: Number of Cases in All Possible Subgroups

Main Group	Gender	CCA Side					
		Right		Left		Any	
		S	NS	S	NS	S	NS
RA / no narr.	Male	1	12	1	14	1	12

	Female	1	48	1	54	1	47
RA / narr.	Male	0	2	0	0	0	2
	Female	0	9	0	3	0	10
Healthy / no narr.	Male	9	31	10	32	9	30
	Female	0	29	0	34	0	29
Healthy / narr.	Male	1	2	1	1	1	3
	Female	0	6	1	1	0	6
